# XLH Matters 2022: Insights and recommendations to improve outcomes for people living with X-linked hypophosphataemia (XLH)

**DOI:** 10.1186/s13023-023-02883-3

**Published:** 2023-10-27

**Authors:** Lothar Seefried, Ali Alzahrani, Pedro Arango Sancho, Justine Bacchetta, Rachel Crowley, Francesco Emma, Jonathan Gibbins, Anna Grandone, Muhammad Kassim Javaid, Gabriel Mindler, Adalbert Raimann, Anya Rothenbuhler, Ian Tucker, Leonid Zeitlin, Agnès Linglart

**Affiliations:** 1 Orthopedic Institute, König-Ludwig Haus, University of Würzburg, Würzburg, Germany; 2 King Faisal Specialist Hospital and Research Center, Riyadh, Saudi Arabia; 3 Department of Pediatric Nephrology, Hospital Sant Joan de Déu, Barcelona, Spain; 4 Department of Onco-Nephrology, Pediatric Cancer Center, Barcelona, Spain; 5 Pediatric Nephrology, Reference Center for Rare Diseases of Calcium and Phosphate, Filières OSCAR et ORKID, INSERM1033, Hospices Civils de Lyon, Lyon, France; 6 St Vincent’s University Hospital and Rare Disease Clinical Trial Network, University College Dublin, Dublin, Ireland; 7 Division of Nephrology, Children’s Hospital Bambino Gesù–IRCCS, Rome, Italy; 8 Evelina London Children’s Hospital, London, UK; 9 Department of Woman, Child, General and Specialized Surgery, University of Campania L. Vanvitelli, Naples, Italy; 10 Nuffield Department of Orthopaedics, Rheumatology and Musculoskeletal Sciences, University of Oxford, Oxford, UK; 11 Department of Pediatric Orthopaedics, Orthopaedic Hospital Speising, Vienna, Austria; 12 Vienna Bone and Growth Center, Vienna, Austria; 13 APHP, Endocrinology and Diabetology for Children, Bicêtre Paris Saclay Hospital, Le Kremlin-Bicêtre, France; 14 APHP, Reference Center for Rare Disorders of Calcium and Phosphate Metabolism, Filière OSCAR, Paris, France; 15 APHP, Platform of Expertise for Rare Disorders Paris Saclay, Bicêtre Paris Saclay Hospital, Le Kremlin-Bicêtre, France; 16 Bristol Royal Hospital for Children, University Hospitals Bristol and Weston NHS Foundation Trust, Bristol, UK; 17 Pediatric Bone Clinic, Orthopedic Department, Dana-Dwek Children’s Hospital, Tel-Aviv Medical Center, Tel Aviv-Yafo, Israel

## Introduction to X-linked hypophosphataemia (XLH) and XLH Matters

To establish clear recommendations for the optimal treatment of people with XLH from adolescence to adulthood, existing guidance needs to be regularly reviewed and updated with real-world experience and recently published long-term trial data for burosumab, taking into account patient-centred outcomes. However, this takes time and interim solutions are needed to provide practical solutions to support healthcare professionals (HCPs) in standardising and improving the overall management of patients with XLH.

Compilation of real-world data and sharing of best practice to guide treatment decisions for people with XLH is encouraged. Due to the progressive nature of this field, we advise clinicians who work in XLH to continuously use networking meetings, such as XLH Matters, as a platform to share their experiences and gain up-to-date insights into current knowledge between publications of updated written guidance. By doing so, clinicians can exchange valuable regional knowledge that can benefit their international XLH colleagues and help to improve outcomes for people with XLH.

### Background

XLH is a rare, genetic disorder associated with a diverse range of clinical manifestations in both children and adults [1]. The disease is progressive and lifelong, with clinical symptoms usually developing during the first or second year of life and proceeding over time, into adulthood [2–4].

For people living with XLH, chronic hypophosphataemia has a multisystemic effect causing deterioration of the bones, teeth, muscles, joints and hearing [4–6]. Clinical manifestations in children include impaired growth, spontaneous dental abscesses, rickets leading to bone deformities, gait abnormalities, craniosynostosis, pain and impairment in physical function and mobility [1–3]. Some manifestations such as lower limb deformity and growth deviations may become worse in adulthood if they are not effectively managed in childhood [4]. In addition to the consequences of unresolved childhood disease, with persistent hypophosphataemia, adults with XLH are at risk of further complications, including early-onset osteoarthritis, osteomalacia, pseudofractures, enthesopathies, spinal stenosis, hearing loss, hyperparathyroidism, obesity and nephrocalcinosis; all of which can impact their health-related quality of life (HRQoL) [2, 5, 6].

People with XLH experience an accumulation of symptoms and complications as they enter different life stages, leading to an increase in the burden of disease. Moreover, some therapies used to prevent the development of rickets (e.g. oral phosphate and active vitamin D analogues) can induce burdensome complications. In children, the burden is due to the immediate consequences of the mineral disorder and the associated consequences of low phosphate on musculoskeletal development. This burden is encumbered further by the need for multiple daily doses of conventional therapy with side effects that can lead to poor adherence to the treatment regimen [3, 7]. In adolescents, the burden of disease is multifactorial and includes the psychological impact of the disease [3, 7]. In adults, the burden includes chronic pain and physical function limitation that results in decreased mobility [7]. The decline in HRQoL places a considerable burden on the mental wellbeing of people living with XLH, putting them at a higher risk of depression and socioeconomic deprivation than the general population [8].

Due to the multisystemic, progressive nature of the disease and associated burden, people living with XLH require lifelong care from a range of multidisciplinary specialists [2]. It is preferable to have a lead clinician, ideally with expertise in metabolic bone diseases, to lead and coordinate the care of a person living with XLH, working with rheumatologists, endocrinologists, nephrologists, dentists, psychologists, orthopaedic surgeons, ear, nose and throat (ENT) specialists, physiotherapists and neurologists, as and when required [2, 6]. However, anecdotal reports indicate that limited availability and access to specialists, and a lack of awareness of XLH among clinicians, have delayed the establishment of multidisciplinary care in some regions and countries. Delayed diagnosis has been shown to exacerbate the burden of disease in people with XLH. Increased awareness of the clinical features of XLH by primary care physicians is essential for prompt referral to an expert centre specialising in bone and mineral diseases [6].

The rarity of XLH can make it difficult for adults and the families of those with XLH to locate clinicians familiar with XLH in any capacity, and much less its manifestation in adult patients [3, 9]. It is important that clinicians within the multidisciplinary team recognise the evolving burden on HRQoL and collaborate to tailor disease management approaches throughout the lifetime [9, 10] of a person living with XLH.

### Overview of XLH Matters 2022

At the XLH Matters 2022 meeting (Box 1), the expert faculty focused on three core topics:

1. The challenges of managing the spectrum of clinical manifestations associated with XLH
2. Identifying treatment goals
3. Optimising the transition of people living with XLH from paediatric to adult healthcare services

**Box 1:** What is XLH Matters?

XLH Matters is an international, annual networking event for clinicians working in XLH across a range of specialities and geographical regions. The XLH Matters series is a forum for international XLH expert clinicians to: share latest clinical research, including real-world data; discuss challenges with managing XLH; and identify approaches to improve the outcomes of people living with XLH. The inaugural XLH Matters was held virtually in 2021, with the second meeting held in person in Madrid in October 2022. At XLH Matters 2022, there were 73 in-person attendees representing 18 countries across Europe and the Middle East. The third meeting was planned in June 2023 in Frankfurt and will build on topics discussed during the previous meetings	

Through structured workshop discussions recorded on workmats (Table 1), the challenges and unmet needs were captured, highlighting where more data, research and education are needed to improve treatment plans, transition to adult care and access lifelong, multidisciplinary specialists. The three core topics were also explored in separate workshop sessions. The key findings from these workshop sessions and roundtable discussions during XLH Matters 2022 are summarised in this supplement.

**Table 1:** XLH Matters 2022 workshop sessions

**Workshop 1: The multiple clinical manifestations of XLH**	
Part 1	Attendees were split into 13 groups and asked to list all children, adult and overlapping manifestations of XLH
Part 2	Attendees were asked to identify the XLH manifestations that, as a group, they considered to be well known, commonly missed and challenging in their clinical practice
**Workshop 2: Establishing treatment goals for people living with XLH**	
Part 1	Attendees were split into 13 groups^†^. In their groups, attendees listed the treatment goals for patients and healthcare professionals
Part 2	Attendees were then asked to identify the treatment goals that, as a group, they considered to be (1) most important and (2) most challenging
**Workshop 3: Challenges of transition from paediatric to adult care**	
Part 1	Attendees were split into 13 groups and asked to select their top 3 transition challenges, as a group
Part 2	Co-chairs facilitated a discussion with all attendees and established the top 10 transition challenges
Part 3	Attendees then voted individually for their highest-ranking transition challenge

^†^Note for workshop 2 only, the 13 groups were split into 11 paediatric-treating and 2 adult-treating groups

### Aim of this supplement

The first comprehensive clinical practice recommendations to support clinicians in the diagnosis and management of XLH were published in 2019 [2]. However, following the publication of these recommendations, further clinical trial data and real-world experience concerning the practical implementation of optimal patient care have become available.

This supplement aims to summarise discussions from the XLH Matters 2022 meeting to support ongoing knowledge sharing, real-world challenges and locate gaps for future research/publication in the XLH field. Consequently, all clinicians working in XLH are encouraged to proactively share their real-world clinical experiences of treating XLH to support upcoming clinical practice recommendations and, ultimately, shape the future care for people living with XLH.

## The multiple clinical manifestations of X-linked hypophosphataemia (XLH)

### Background

**Fig. 1:**
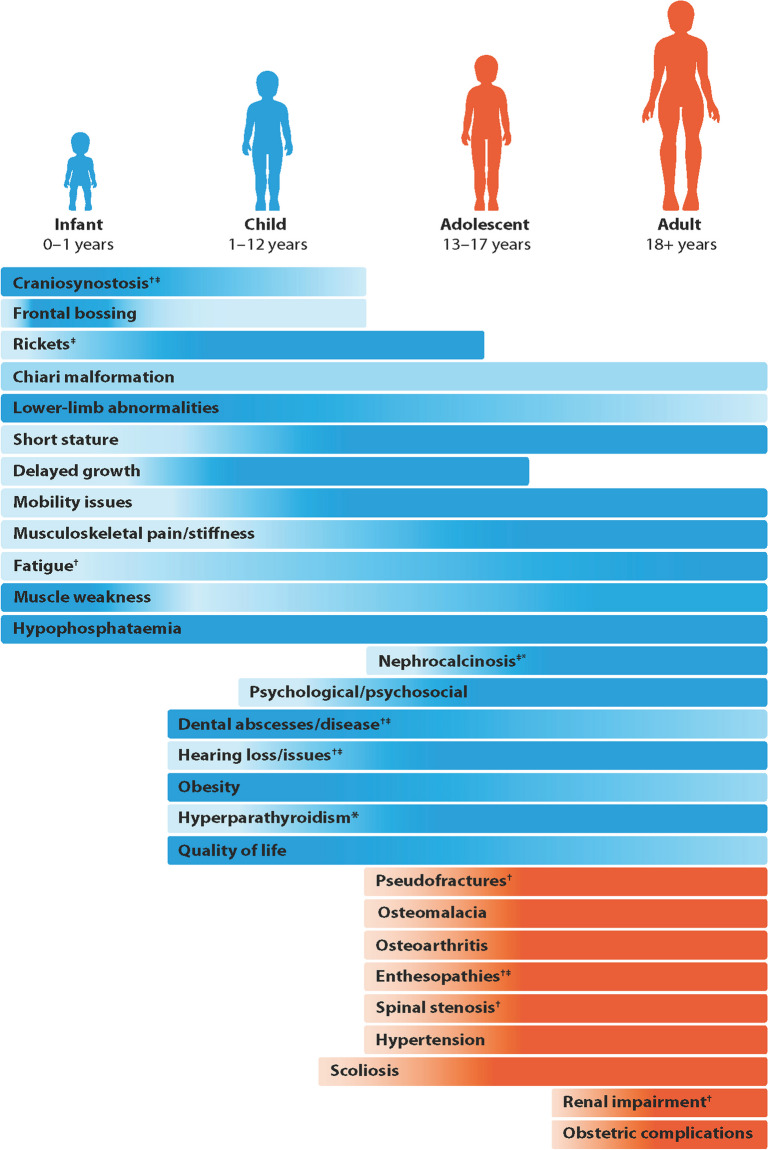
Drawing the clinical picture. The clinical manifestations that present throughout the lifespan of people living with XLH as discussed by the attendees at XLH Matters. Blue indicates the clinical manifestations that first present in early childhood, while orange represents those that manifest in adolescence and adulthood. An increase in colour scale denotes an increasing burden of individual manifestions. ^†^Clinical manifestations identified as commonly missed in patients with XLH; ^‡^Clinical manifestations identified as challenging to manage in patients with XLH; *Clinical manifestations associated with conventional therapy

People living with XLH experience a diverse range of often debilitating clinical manifestations associated with XLH [2, 4, 11]. These manifestations present and accumulate at different stages throughout their lifetime, leading to an often underestimated burden of disease (Fig. 1). Moreover, the course and severity of clinical manifestations can differ between individuals, highlighting the phenotypic spectrum of the disease and the need for individually optimised clinical management of XLH [2].

Clinical manifestations of XLH usually present in early childhood, with chronic hypophosphataemia leading to the development of rickets, disproportionate growth and limb deformities that predominantly affect the lower limbs. Consequently, children with XLH commonly experience delayed motor development, gait abnormalities and pain, resulting in corrective limb surgeries in up to two-thirds of children in historical cohorts during or at the end of growth [2–4, 12]. Additional clinical challenges in children include craniosynostosis and dental abnormalities, such as recurrent dental abscesses [12, 13]. The accumulation of these clinical manifestations places a substantial disease burden on children living with XLH, having a considerable impact on their emotional wellbeing and HRQoL [4].

Conventional therapy, comprising oral phosphate and active vitamin D analogues, insufficently improves specific clinical manifestations associated with XLH, including growth velocity, lower limb alignment and dental health [2]. Indeed, cohort data show that adults commonly experience unresolved complications of childhood disease due to insufficient action of conventional therapy or absence of treatment during childhood [2, 14]. For example, short stature, as well as lower limb abnormalities, are reported in both children and adults with XLH, with data suggesting that early intervention may improve these manifestations [15–17]. Furthermore, conventional therapy has been reported to produce positive outcomes in the dental health of people with XLH; therefore, poor oral health may be largely associated with a lack of access to appropriate treatment due to the rarity of the disease [14, 18]. The incidence of obesity is increased in children and adults with XLH compared with the general population, owing partly to chronic skeletal abnormalities and impaired mobility [2]. Oral phosphate and active vitamin D analogues do not appear to improve hearing loss, with hearing impairments such as episodic tinnitus and deafness becoming increasingly frequent with advancing age in adults with XLH [2]. Moreover, long-term use of conventional therapy is associated with decreased adherence, persistent osteomalacia and rickets, as well as an increased risk of complications such as hypercalciuria, nephrocalcinosis and hyperparathyroidism [2, 4, 10, 11]. Thus, inadequate monitoring of these complications leads to further clinical manifestations and impacts HRQoL [10].

In addition to the consequences of unresolved complications of childhood disease, adults with XLH experience further clinical manifestations, consistent with chronic hypophosphataemia and the progression of osteomalacia (Fig. 1). Notably, musculoskeletal features, including pseudofractures, enthesopathies, osteoarthritis (OA), and spinal stenosis become increasingly common with age and significantly contribute to pain, joint stiffness and muscle weakness in adults living with XLH [4, 19].

### An underestimated burden of disease

The burden caused by XLH is often underestimated, with multiple surveys highlighting the substantial burden experienced by people living with XLH, regardless of age [4, 19]. Pain, muscle weakness and impaired physical function are frequently reported in both children and adults with XLH and considerably impact their HRQoL [4, 12]. Of note, mean patient-reported outcome scores evaluating physical function were considerably worse in people living with XLH than those in the general population [4]. For example, mean physical function scores derived from the Western Ontario and McMaster Universities Osteoarthritis Index (WOMAC) were considerably higher (i.e. worse) in adults with XLH at 40.8 compared with the general population score of 15.4, indicating an inferior physical function [4]. HRQoL measures assessing pain and physical component scores were also significantly worse for adults with XLH compared with those living with axial spondyloarthritis, another systemic skeletal disease known to affect HRQoL [20].

A qualitative analysis of survey responses received from people living with XLH during the 2018 National Institute for Heath and Care Excellence (NICE) public consultation provided further important insights into the progressive impact of XLH [7]. In children, limitations associated with conventional therapy and pain were frequently identified as the greatest burden. For adolescents, the psychological burdens of XLH were dominant, with >25% of statements related to these impacts [7]. In adults, the burden of persisting complications remained; however, pain, mobility issues and concerns regarding access to appropriate treatment were commonly stated [3, 7]. Together, these findings highlight the evolving nature of the disease burden associated with XLH.

### XLH Matters 2022 discussion

The challenges in managing the wide spectrum of clinical manifestations associated with XLH were addressed during the first workshop at the XLH Matters 2022 meeting. Attendees discussed the most common, commonly missed and challenging clinical manifestations associated with XLH, with their responses recorded on workmats (see summaries in Boxes 2, 3 and 4).

**Box 2:** The multiple clinical manifestations of XLH experienced by children and adults with XLH

• All attendees listed short stature and dental abscesses/disease as a common manifestation of XLH in children and adults	
• Most attendees listed dental abscesses/disease, hearing loss, chronic pain and psychological/psychosocial manifestations as commonly missed manifestations, with many also finding it a challenging manifestation to manage in both children and adults	
• Other commonly missed manifestations in children and adults included obesity and scoliosis

**Box 3:** The multiple clinical manifestations of children with XLH

• Craniosynostosis and delayed growth were both identified as commonly missed and challenging manifestations to manage in children	
• Attendees identified rickets as a challenging manifestation to manage in children

**Box 4:** The multiple clinical manifestations of adults with XLH

• Enthesopathies were identified as a commonly missed manifestation and the most challenging to manage in adults	
• Other commonly missed manifestations in adults included spinal stenosis and hypertension, and pseudofractures, renal impairment and obstetric complications were agreed to be the most challenging to manage	

The most common manifestations of XLH were acknowledged during this workshop activity, whereas the roundtable discussions focused on those most commonly missed and challenging to manage, with attendees highlighting that it is important to raise awareness of the full range of manifestations associated with XLH. The evolving burden of XLH was also noted by attendees, with calls for treatment strategies to minimise the development of complications arising from XLH itself and related interventions. The evolving burden of XLH manifestations as discussed during this workshop are further summarised in Fig. 1.

#### Importance of a multidisciplinary approach

The importance of a multidisciplinary treatment approach is increasingly being recognised given the diversity of clinical manifestations that continue to present at different stages throughout the lifetime of people living with XLH [2, 11]. Attendees supported this consensus, noting the paramount importance of establishing a network of multidisciplinary specialists to aid the management of manifestations, as required, and to optimise care of people living with XLH. Where possible, this network should be coordinated by a lead clinician, ideally a metabolic bone specialist, who engages with neurologists, endocrinologists, rheumatologists, dentists, orthopaedic surgeons, ear nose and throat specialists (ENTs), physiotherapists, neurologists and psychologists throughout the lifetime of people living with XLH, as and when needed [2, 10, 11]. Regular, structured follow-up visits with a metabolic bone specialist are necessary to ensure early intervention and management of potential complications as the disease progresses [11].

### Concluding remarks

The findings from this XLH Matters 2022 workshop encourage concerted and coordinated efforts to establish long-term treatment recommendations to optimise the clinical care of people living with XLH. These recommendations should incorporate clinical advancements to support the development of universally standardised assessments relating to the diagnosis and treatment of XLH, including assessment across the different clinical manifestations. It is essential for new recommendations to outline strategies regarding routine follow-up visits to monitor the progression and impact of XLH. In addition, targeted measures to manage challenging manifestations beyond the treatment of XLH itself are also increasingly necessary and require attention. Access to a multidisciplinary support network and comprehensive strategies will aid widespread cross-communication between different centres, leading to optimal outcomes for people living with XLH.

## Establishing treatment goals for people living with X-linked hypophosphataemia (XLH)

### Background

The disease burden evolves throughout the lifespan of people living with XLH, and different clinical manifestations occur at different ages in varying ranges of severity [2, 7]. Due to the symptom progression over time, treatment goals are tailored to key stages of life: infancy, childhood, adolescence, pregnancy, adulthood and elderly [2, 7]. Treatment goals support clinicians to determine how effective interventions are in reducing the overall disease burden in people living with XLH [21] (Box 5).

**Box 5:** Markers of treatment efficacy in XLH

1. Biomarkers (e.g. alkaline phosphatase levels)	
2. Clinical manifestations (e.g. lower limb deformities, linear growth)	
3. Imaging (e.g. pseudofracture healing, deformity analysis)	
4. Patient-reported outcomes (e.g. pain)	
These markers depend on the age of the person living with XLH and status of their disease [2, 7, 21]	

### Defining treatment goals in XLH

Treatment goals are typically consistent with halting the progression of the clinical manifestations and development of new symptoms, which require regular monitoring to ensure optimal disease management [21]. Treatment goals can be categorised into:

1. *Short-term goals* that aim to assess the restoration of phosphate homeostasis, the immediate impact of interventions and mitigate immediate symptoms, e.g. pain, fatigue, physical function and HRQoL [12]
2. *Long-term goals* that improve patient outcomes by attaining the best possible final height, reducing long-term complications, and improving physical function and wellbeing [12, 21]

Together, these goals aim to improve HRQoL by feeding into the overarching objective of halting disease progression by targeting the underlying pathogenesis of XLH. As there are differences between the treatment goals among children, adolescents and adults, people living with XLH need an individualised treatment approach that considers their long-term outcomes. During the lifetime of people living with XLH, treatment goals should be reviewed in the context of the medical history, needs of the patients, evolution of the disease and social environment.

Before diagnosis, a child with XLH will often present with the aforementioned clinical manifestations when they first make contact with the clinician (Fig. 1) [2, 21]. When the diagnosis of XLH is confirmed, the initial treatment goal in children typically will be healing of rickets, measured by radiological assessments, and decrease of alkaline phosphatase levels [21]. Following the healing or attenuation of rickets, treatment goals in children will often focus on increasing growth potential, reducing bone pain, correcting leg deformities, improving muscle function and overall dental health [5, 22]. Some children with XLH are treated with the traditional conventional treatment, with multiple daily doses of phosphate and active vitamin D analogues to counteract persistent renal phosphate wasting and downstream effects of excess fibroblast growth factor 23 (FGF23) [5, 14, 22]. However, this treatment approach does not address the underlying pathogenesis of XLH and, therefore, does not restore phosphate homeostasis or fully halt disease progression [12, 14]*.*

The definitive long-term treatment goal for people living with XLH is to optimise outcomes throughout life, irrespective of age or clinical manifestations, by improving phosphate homeostasis [23, 24]. Interventions such as burosumab, which effectively improve phosphate homeostasis by counteracting the effects of the FGF23 signalling pathway, can support people with XLH to reach their long-term treatment goals. Early and individually tailored therapy and referral to a multidisciplinary team (MDT) helps to prevent the development of long-term complications and improve the HRQoL of people with XLH [2, 5, 17, 21, 22].

#### XLH Matters 2022 discussion

**Fig. 2:**
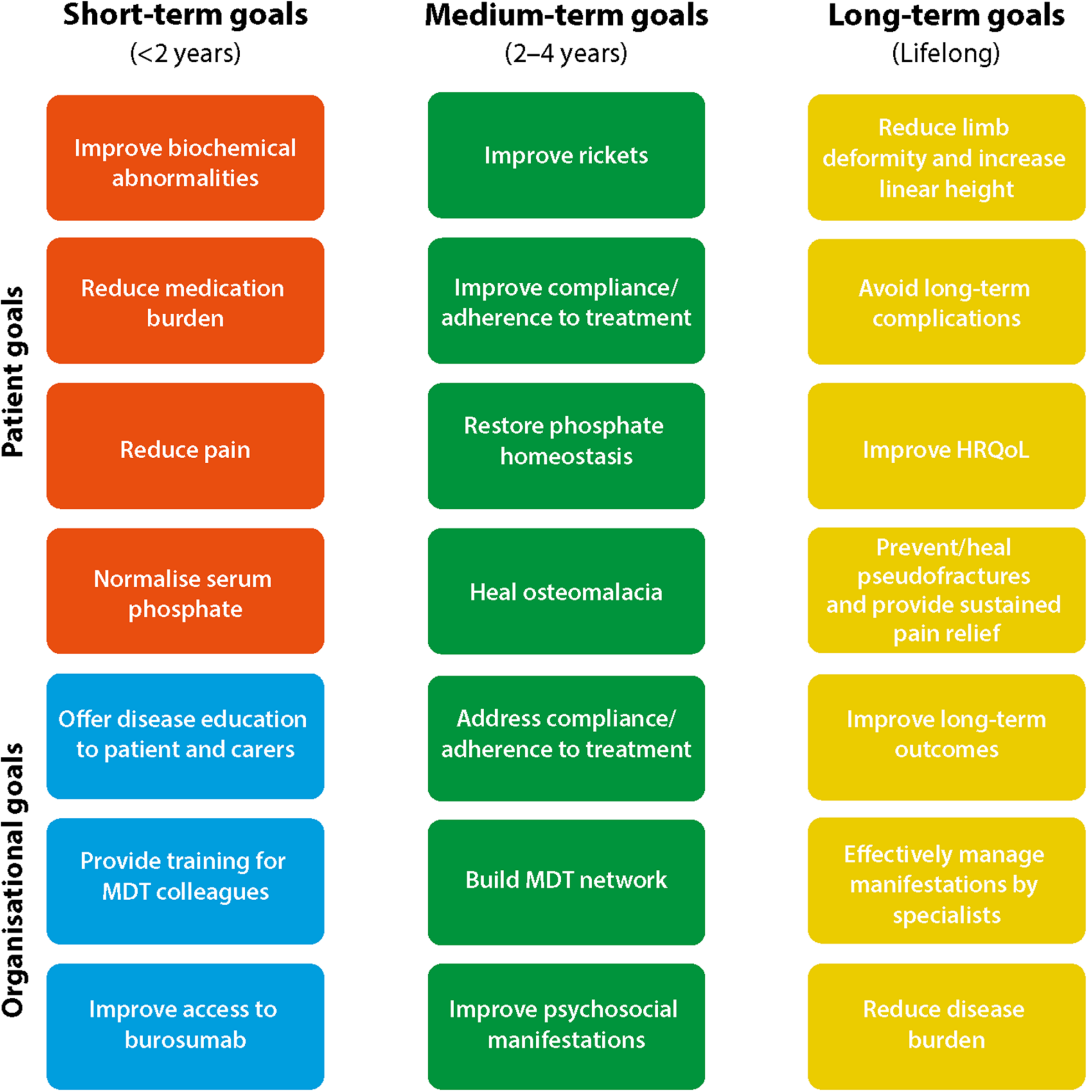
Recommended achievable goals for people living with XLH. Real-world clinical examples of short, medium and long-term patient goals discussed by XLH specialists at the XLH Matters 2022 meeting. Achieving short-term goals, in turn, leads to achieving medium and long-term outcomes. HRQoL, health-related quality of life; MDT, multidisciplinary team

The focus of the second workshop session at XLH Matters 2022 was establishing achievable treatment goals for people living with XLH. Attendees discussed appropriate treatment goals for young children, adolescents and adults with XLH, with outputs recorded on workmats. The discussion focused on the importance of setting achievable and measurable goals, in addition to considering the differences between short-, medium- and long-term goals. The output of the discussions is summarised in Fig. 2, reflecting how achieving short-term goals will ultimately lead to achieving medium- and longer-term goals.

For example, when treating children with XLH, a short-term treatment goal is to minimise phosphate-wasting, indicated by the normalisation of biochemical abnormalities, thereby healing rickets to reduce long-term outcomes, i.e. bowed legs and impaired growth. Short-term treatment goals in adults are normalising serum phosphate and healing osteomalacia to improve long-term outcomes, i.e. prevention/healing of fractures and relief from bone pain.

Unsurprisingly, and in line with published literature, improving growth and deformities were the highest ranked treatment goals in young children [5]. However, in adolescents, there was a greater focus on treatment goals related to HRQoL and psychosocial manifestations, with the latter listed as the most challenging to achieve. In adolescents, the burden of disease becomes increasingly complex, with a greater physiological impact placing people with XLH at higher risk of mental illness and socioeconomic deprivation [7, 8]. Attendees at XLH Matters 2022 emphasised the ‘perception of appearance’ in adolescent treatment goals. During discussions, attendees suggested that adolescents are more aware of the impact of living with XLH than younger children, and are conscious of being differentiated from their peers, which often impacts their HRQoL.

All adult-treating attendees agreed that improving pain, fatigue and HRQoL are the ‘most important’ and ‘most challenging’ adult treatment goals, supporting discussions at the meeting that stressed the impact pain and fatigue have on HRQoL in adults with XLH. Some people with XLH require holistic treatments, such as physical therapy, to relieve the progressive manifestations of XLH, depending on their severity [2]. The aims of physical therapy include providing pain relief, improving physical function and fitness, and reducing XLH-related disability [2]. This is another example of how short-term goals support the long-term goal of improving HRQoL. Adult-treating attendees also indicated the importance of sexual health and family planning as treatment goals, emphasising the lifelong impact of XLH on HRQoL.

Adult-treating attendees also highlighted that establishing an MDT is the ‘most important’ treatment management goal for healthcare professionals, supporting published recommendations that people living with XLH should receive lifelong care from multidisciplinary specialists [5]. Many XLH treatment goals span across all age ranges; interventions at a younger age impact how achievable long-term goals are later in a person’s life. Moreover, sub-optimal disease management during childhood can worsen many XLH complications in adults rendering some long-term treatment goals, such as increasing growth potential and preventing lower limb deformities, unachievable [4]. The association between early intervention in children with XLH and optimised long-term outcomes is emphasised in patients with delayed diagnosis or misdiagnosis, who typically receive insufficient treatment interventions [4, 25]. Earlier diagnosis and initiation of treatment are critical short-term goals that will impact the longer-term outcomes of people living with XLH [22].

#### Agreeing individual care plans

Attendees stressed the unique perspective of each person living with XLH, stating that individual priorities and clinical needs are both important for disease management plans. For example, as people with XLH get older, they could become increasingly aware of the future implications of living with XLH in the short term (limitations to physical activities with peers) and long term (future impact on work or starting a family) [26, 27]. In addition, the disease manifests differently over the lifespan, so treatment goals may change as a person with XLH ages. Therefore, the lead clinician and person with XLH need to agree on the long-term goals of treatment to establish a mutual understanding of how they can align their approaches to achieve optimal long-term outcomes, but also regularly review the goals in case these need to be changed. Moreover, if treatment goals are discussed and aligned with people with XLH and their families, they can actively endeavour to achieve them [27].

### Concluding remarks

It is necessary for clinicians to regularly assess and adjust treatment goals throughout the lifetime of people living with XLH. We propose that clinicians working with patients living with XLH outline short-term treatment goals, which consider how XLH can progress throughout the lifetime. All short-term treatment goals should form part of the broader plan for the lifelong management of a person with XLH.

## Challenges of transition from paediatric to adult care in X-linked hypophosphataemia (XLH)

### Background

Adequate transition from paediatric to adult healthcare services is essential to ensure effective lifelong management of many paediatric-onset diseases, including XLH [6]. However, the transition process presents various challenges, with adolescents often lost to follow-up care, negatively impacting their health outcomes in adulthood [28]. Discussions at XLH Matters 2022 highlighted the importance of preparing for transition early, with the process ideally being introduced at 12–13 years of age. The advantages of continued collaboration between paediatric and adult healthcare services following transfer were also noted by attendees. Given the importance of continued care in XLH management, tailored healthcare transition recommendations incorporating the individual needs of adolescents with XLH would facilitate optimal transition from paediatric to adult services. This section explores the challenges associated with the transition process and includes discussion from XLH Matters 2022 regarding real-life transition challenges, and proposes strategies to aid successful transition of adolescents with XLH.

### Transition challenges

Transition of care occurs at a critical period in the lives of adolescents, with the likely progressive accumulation of clinical manifestations into adulthood, placing an additional physical and emotional burden on adolescents with XLH and their families (Fig. 1) [4, 7, 29]. While XLH presents differently throughout the lifetime of individuals, adolescents may experience an emergence of additional musculoskeletal symptoms [19, 30]. Therefore, education from experts is required to ensure adolescents are aware of the progressive nature of XLH and how this may affect them individually [4]. Adolescents may also experience anxieties related to XLH, in addition to typical worries associated with adolescence. For example, the psychological impact of XLH becomes prominent in adolescents as they become increasingly aware of the physical limitations associated with their lifelong condition [7]. Furthermore, adolescents living with XLH are establishing independence, which includes crucial decision-making regarding their education and career [29]. Therefore, access to educational resources and optional psychosocial support during this period is important to aid transition.

### Existing recommendations

General healthcare transition guidelines for chronic diseases and other rare diseases, such as osteogenesis imperfecta (OI), another rare metabolic bone disorder, are available; however, guidelines specifically relating to the transition of adolescents with XLH do not exist [28, 31]. Given the similarities between OI and XLH, the OI guidance may provide a good basis for establishing healthcare transition strategies for adolescents with XLH [28]. Indeed, established concepts for OI highlighted important topics including disease awareness and self-advocacy, areas which were identified as support gaps in a recent XLH expert consensus report [28, 31]. This report provided the first expert recommendations for the transition of adolescents with XLH, outlining three areas for improvement: foundational knowledge, information transfer and supporting engagement [31]. While these recommendations provide a firm starting point, further research and support is needed to develop tailored XLH healthcare transition guidance.

### XLH Matters 2022 discussion

**Fig. 3:**
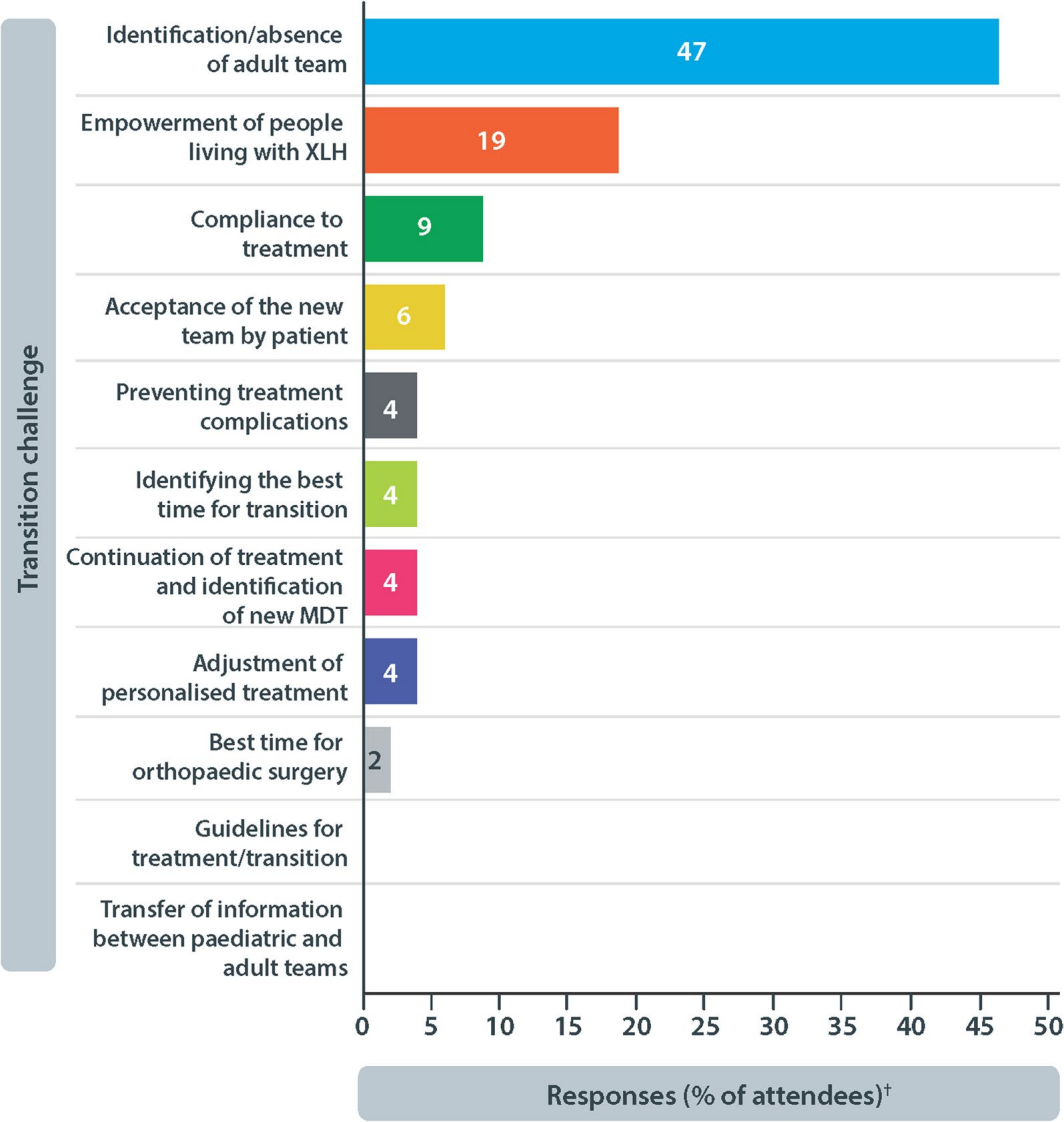
Challenges associated with transition from paediatric to adult care. Attendees at XLH Matters 2022 selected their primary transition challenge from a list of challenges identified during the workshop session. ^†^Based on 47 responses. MDT, multidisciplinary team; XLH, X-linked hypophosphataemia

To understand the challenges associated with transition of adolescents with XLH in clinical practice, attendees discussed commonly encountered transition challenges during a workshop session at XLH Matters 2022. The attendees created a list of their top transition challenges before individually voting for their primary challenge. The key challenges identified by the attendees are summarised in Fig. 3.

The identification/absence of an adult team was the most frequently reported transition challenge (47% of attendees, 22/47). XLH has often been considered a paediatric disease so identifying adult HCPs with experience of managing XLH can be difficult [28]. Attendees also emphasised the need to expand the number of multidisciplinary specialists available within adult healthcare services. Raising awareness of XLH among HCPs treating adults and increasing the number of HCPs specialising in XLH management will address some of the gaps in establishing multidisciplinary team (MDT) networks. Early collaboration between paediatric and adult HCPs during the transition process, will provide adult HCPs with in-depth insights on the specific needs of the adolescents transitioning to their care, ensuring tailored management. Given the central role of continued care in the management of XLH, attendees agreed support must be given to primary coordinators/paediatricians to identify and establish long-term partnerships with adult HCPs.

The second biggest challenge in the transition process was ‘empowerment of people living with XLH’ voted by 9/47 (19%) attendees. While the optimal age to introduce the transition process varied regionally, attendees agreed that open communication from an early age supports successful transition. Early preparation facilitates a gradual transfer of ‘disease ownership’ and encourages adolescents to be active contributors to their care. The importance of educating young people about their condition was highlighted, with a focus on explaining the progressive nature of XLH. With this knowledge, adolescents with XLH can appreciate the benefits of pro-active management on their future health outcomes. To promote their engagement, the development of educational resources utilising communication channels familiar to adolescents are needed, such as social media platforms and online portals. Providing additional support to the parents or carers of those transitioning to adult services, including access to guidance on empowering their child as they mature, is also vital.

Attendees acknowledged the importance of revisiting transitional planning milestones regularly throughout the healthcare transition process. This ensures that the changing needs of individuals are being met and any gaps in transition readiness are promptly addressed. Of note, attendees suggested several useful tools to assess transition readiness, including transition readiness assessment questionnaires (TRAQ) (available online) and the Ready, Steady, Go programme (available online).

Concerns regarding adherence to treatment in adolescents have previously been reported [32]. Therefore, it is unsurprising that attendees voted compliance to treatment as a challenge. Given the importance of improving HRQoL outcomes, strategies that encourage compliance to treatment are required. For example, efforts to alleviate certain anxieties associated with transfer to adult services may prove useful in maintaining adherence. Attendees noted the significance of shared visits prior to transfer, as well as disease awareness. Increasing the confidence of adolescents to manage their condition and engage with adult HCPs builds a good foundation for a successful transition. Encouraging continued collaboration between paediatric and adult services is paramount, and the attendees highlighted the benefits of a primary contact to support adolescents during and after transition.

Further discussions at XLH Matters 2022 noted the challenges of continuing treatment in adolescents living with XLH. Attendees mentioned that treatment with oral phosphate and active vitamin D analogues is often discontinued upon completion of growth, with treatment resumed in adulthood based on the presentation of symptoms. These gaps in care contribute to the increasing disease burden experienced by people living with XLH, which is discussed in the next section – Exploring access to treatment for people with X-linked hypophosphataemia (XLH).

### Optimising the transition process

The importance of continuity of care in managing chronic diseases means that healthcare transition programmes have been effective in minimising loss to follow-up in adulthood [31]. Adolescents lost to follow-up are unlikely to access the care that they need during adulthood, negatively impacting their HRQoL [28, 31]. The development of healthcare transition programmes have resulted in positive outcomes during the transition of those living with common and rare chronic diseases, including increased adherence to treatment and engagement with ambulatory services [28, 31]. All attendees indicated their support for the development of practical concepts that enable a flexible approach to transition, with regional centres adopting strategies that work for them and the individuals in their care. This will improve the transition of adolescents to adult service, to secure lifelong expert management for people living with XLH.

### Concluding remarks

At XLH Matters 2022, there was a clear call to action to develop adaptable healthcare transition recommendations that incorporate the specific needs of young people living with XLH and promote tailored practices across different regional centres. Expert recommendations regarding transition from paediatric to adult services, as discussed at XLH Matters 2022, are summarised in Box 6. Three key factors for establishing successful transition were: (1) identifying an engaged adult HCP team; (2) continued collaboration between paediatric and adult services; and (3) encourage adolescents to actively participate in their care through disease education and the development of self-management skills. Optimising transition facilitates adolescents to access the specialist care that they require during adulthood, leading to improved HRQoL for both young people and adults living with XLH.

**Box 6:** XLH Matters 2022: Expert recommendations regarding transition from paediatric to adult care for adolescents living with XLH

• Prepare young people for transition early	
• To promote a gradual increase in disease knowledge and development of self-management skills	
• Utilise technology platforms familiar to young adults to provide educational resources and encourage engagement	
• Families may require additional support to encourage the empowerment of adolescents	
• Assess transition readiness and disease knowledge regularly to continually address gaps	
• Assessment tools are available worldwide, including the Ready, Steady, Go programme and TRAQ	
• Adolescents and their families may require access to psychological support and resources	
• Ensure collaboration between paediatric and adult clinicians to enable comprehensive planning and transition preparation	
• Early communication and transfer of medical information allows adult clinicians to understand the individual needs of those in their care	
• A primary coordinator/point of contact during and after transition can aid successful transfer to adult services	
• Introduce adolescents to their new adult team prior to transfer	
• Shared visits between paediatric and adult services may be beneficial prior to transfer	
• Efforts to verify successful transfer are needed with further opportunities to engage provided if necessary	

## Exploring access to treatment for people with X-linked hypophosphataemia (XLH)

### Background

Treatment practices continuously evolve as the understanding of XLH grows and opportunities for treatment options change. The roundtable discussions at XLH Matters 2022 examined the barriers to manage effectively the spectrum of manifestations associated with XLH. The global community at XLH Matters 2022 highlighted the importance of providing comprehensive guidance that integrates new long-term and real-world burosumab data. New guidance would help establish consistent treatment approaches and provide people with XLH equal access to optimal care. This section explores the variations in access to treatment, including burosumab, and its impact on people living with XLH.

### Burosumab long-term and real-world data

Burosumab is the first disease-modifying therapy approved to treat XLH by inhibiting fibroblast growth factor 23 (FGF23) to restore phosphate homeostasis [2, 23, 33]. A global clinical development programme comprising phase 2 and 3 clinical trials demonstrated the positive efficacy and safety profile of burosumab in children and adults with XLH [23, 24, 34, 35]. In 2018, burosumab received conditional approval from the European Medicines Agency (EMA) for the treatment of XLH in children 1 year of age and older with radiographic evidence of bone disease and adolescents with growing skeletons [23, 33]. By September 2020, with the addition of studies in adults, burosumab had gained full approval from the EMA to treat children and adolescents aged 1‒17 years with radiographic evidence of bone disease and adults with XLH [30].

The clinical practice recommendations published by Haffner et al*.* in 2017 represent the first phase of establishing clear guidance for the diagnosis and management of XLH [2]. These evidence-based recommendations were informed by clinical data available for burosumab. Haffner et al*.* emphasise that, due to the pending data on the cost-effectiveness and long-term outcomes of burosumab at that time, it was not feasible to provide conclusive recommendations on its use in people with XLH [2]. However, since 2018, longer-term and real-world data have become available that supports the long-term safety and efficacy of burosumab for people with XLH [24, 34, 35].

In children with XLH, improvements in phosphate levels and rickets severity score were maintained during 3 years of treatment, and the burosumab safety profile over 160 weeks supported findings from previous paediatric studies [35, 36]. In adults with XLH, long-term use of burosumab demonstrated sustained improvements in biochemical markers, physical function and patient-reported symptoms and HRQoL [37]. In addition, the study found that the benefits of burosumab appear to be lost if treatment is interrupted, but return when treatment is reinstated, advocating the uninterrupted long-term use of burosumab in adults with XLH [37].

Registries provide an effective way of collecting large-scale patient data for long-term assessment of patients [38, 39]. As is often the case for a rare disease, there are limited data on the epidemiology of XLH at an international level [38]. Hence, the International XLH Registry was set up in August 2017, as a non-interventional, observational, real-world data collection programme, enrolling an intent-to-treat population of children and adults with XLH. It is seeking to recruit 1200 participants and run for 10 years [38]. The Registry aims to better understand and improve the lives of people with XLH by building on existing knowledge with real-world evidence to help inform clinical practice [38].

Clinicians are advised to apply an evidence-based approach when making treatment decisions, which includes utilising the evidence from clinical-trial and real-world data integrated with their clinical expertise and the patient’s unique values and preferences.

### XLH Matters 2022 discussion

#### Continuing treatment for adolescents

At XLH Matters 2022, continuing treatment in adolescents with XLH was a recurring topic of discussion, higlighting the uncertainty within the XLH community of how to manage this patient group. Attendees expressed the need for more clarity on optimal treatment regimens for adolescents living with XLH. For example, clinical trials for burosumab enrolled children aged 1–12 years and adults aged 18 years and above but did not enrol adolescents aged 13–17 years [30]. Discussions also indicated that, in many European countries, burosumab treatment is discontinued when longitudinal growth ceases, which can be as early as 12 years of age, especially in girls.

Current published literature reiterates the discussions from the meeting and emphasises that, although the clinical manifestations of XLH persist throughout an individual’s life, in some European countries, the standard clinical practice is to discontinue treatment when skeletal growth is complete [30]. Consequently, when treatment is halted at growth cessation, it is often only resumed when symptoms present in adulthood, resulting in a gap in care [30].

The uncertainty of not being able to continue treatment can cause significant stress for an adolescent living with XLH during a period of already considerable change (e.g. academic exams, finishing school, starting university and leaving home). The clinicians flagged the impact this can have on the HRQoL of adolescents living with XLH. Consequently, attendees stated that there needs to be more guidance on the use of burosumab in older children and adolescents, as well as their management during the transition to adulthood.

The XLH community is actively seeking to share best practice in the management of adolescents as they navigate the complexities of this condition. Since XLH Matters 2022, a group of European clinicians experienced with burosumab treatment in clinical practice published their practice-based insights on the use of burosumab in children and adolescents with XLH [30]. The publication highlighted the lack of clinical studies of burosumab, specifically in adolescents aged 13–17 years, leaving a critical gap in guidance on how to provide optimal care for this subset of patients [30]. The European-based clinical experts endorsed the continuation of burosumab in children throughout adolescence [30]. Their clinical-based insights noted that stopping burosumab treatment conflicts with treatment goals; for example, once treatment is stopped, osteomalacia may progress with the worsening of disease [30]. In line with this, attendees at XLH Matters 2022 identified ‘heal osteomalacia’ as a medium-term treatment goal for adults with XLH (Fig. 2).

The European-based clinical experts and attendees at XLH Matters 2022 reinforced the need for clinical studies and real-world evidence to support the continuation of burosumab in adolescents [30]. Thus, continuing care will now be a specific agenda item at XLH Matters 2023, where attendees can discuss real-world case studies with clinicians experienced with burosumab treatment in adolescents with XLH.

#### Access and reimbursement inequalities

At XLH Matters 2022, regional differences in disease management approaches were flagged as an obstacle preventing some people living with XLH from receiving optimal care. Examples included discontinuation of treatment in adolescents, which were partly due to the gap that can occur between regulatory approval and reimbursement of medicines. Adult-treating clinicians highlighted the variations in treatment reimbursement across different countries, which has led to disparities in treatment access. In many countries in Europe, burosumab is licensed for adults, but it is not reimbursed; however, compassionate use for patients aged >18 years is possible [30]. The regional differences to treatment access can be viewed as an inequality among European citizens, therefore, we are supporting overarching European networks that advocate for equal access to care. The European Reference Network on Rare Endocrine Conditions (EndoERN) and European Reference Network on Rare Bone Diseases (ERN BOND) are two European reference networks for rare diseases that cover XLH in their area of expertise (information avaliable online).

**Fig. 4:**
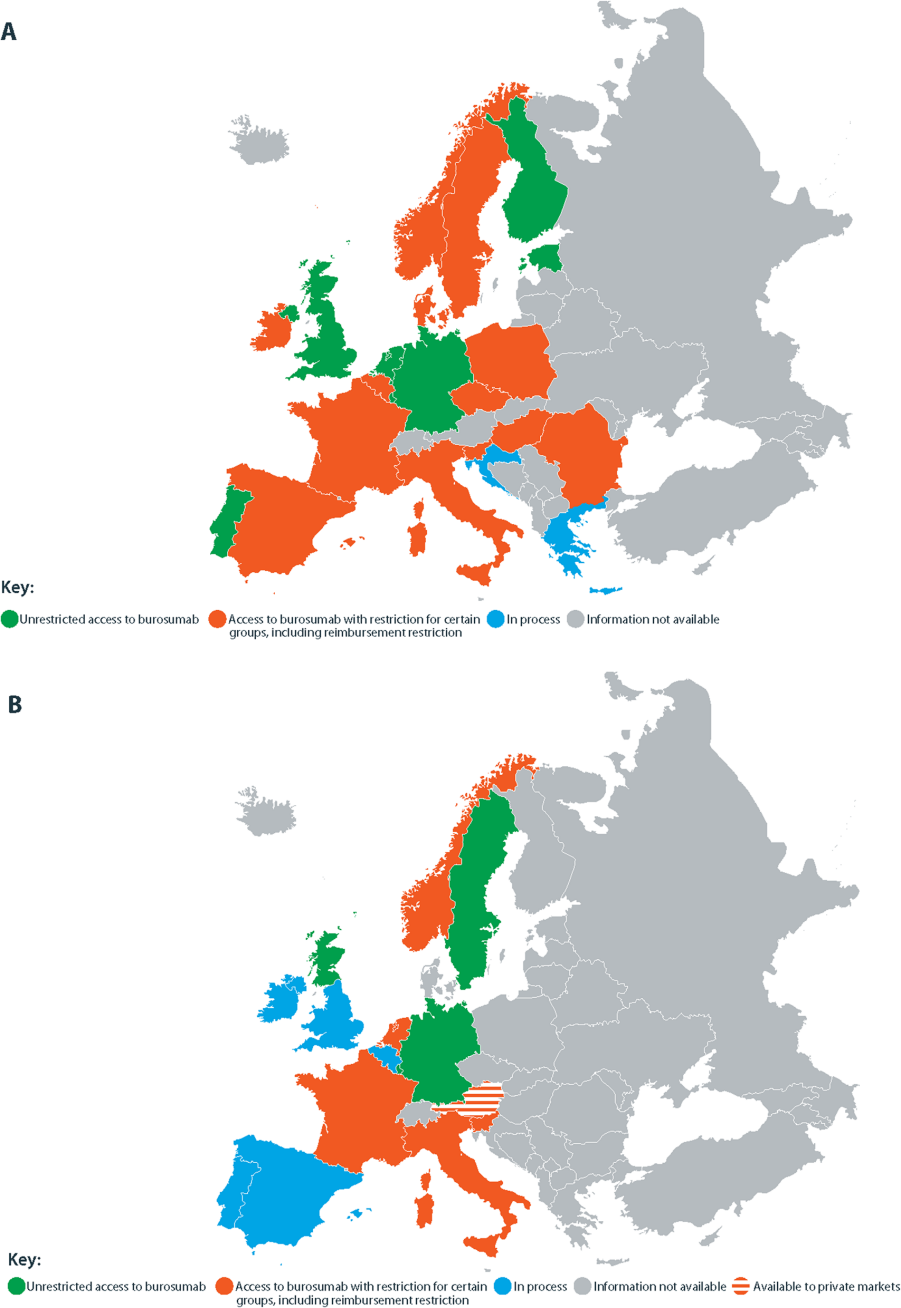
Regional access to burosumab in Europe for people living with XLH^†^. **A** Children with XLH (0–17 years old). **B** Adults with XLH (18+ years old). ^†^As of March 2023

It is evident that there is a variation in the regulatory and reimbursement restrictions (summarised in Fig. 4a and b) for burosumab treatment in different European countries, which impacts treatment in all age groups. Therefore, additional guidance on long-term use of burosumab is imperative to enable changes in regulatory approval and reimbursement policies, to ultimately, reduce variations in access to treatment for people with XLH.

### Concluding remarks

To establish clear recommendations for the optimal treatment of people with XLH from adolescence to adulthood, existing guidance needs to be regularly reviewed and updated with real-world experience and recently published long-term trial data for burosumab, taking into account patient-centred outcomes. However, this takes time and interim solutions are needed to provide practical solutions to support HCPs in standardising and improving the overall management of patients with XLH.

Compilation of real-world data and sharing of best practice to guide treatment decisions for people with XLH is encouraged. Due to the progressive nature of this field, we advise clinicians who work in XLH to continuously use networking meetings, such as XLH Matters, as a platform to share their experiences and gain up-to-date insights into current knowledge between publications of updated written guidance. By doing so, clinicians can exchange valuable regional knowledge that can benefit their international XLH colleagues and help to improve outcomes for people with XLH.
